# EPHA2-dependent outcompetition of KRASG12D mutant cells by wild-type neighbors in the adult pancreas

**DOI:** 10.1016/j.cub.2021.03.094

**Published:** 2021-06-21

**Authors:** William Hill, Andreas Zaragkoulias, Beatriz Salvador-Barbero, Geraint J. Parfitt, Markella Alatsatianos, Ana Padilha, Sean Porazinski, Thomas E. Woolley, Jennifer P. Morton, Owen J. Sansom, Catherine Hogan

**Affiliations:** 1 European Cancer Stem Cell Research Institute, School of Biosciences, Cardiff University, Hadyn Ellis Building, Maindy Road, Cardiff CF24 4HQ, UK; 2 School of Optometry & Vision Sciences, Cardiff University, Maindy Road, Cardiff CF24 4HQ, UK; 3 Faculty of Medicine, St Vincent’s Clinical School, University of New South Wales, Sydney, Australia; 4 School of Mathematics, Cardiff University, Senghennydd Road, Cardiff CF24 4AG, UK; 5 CRUK Beatson Institute, Glasgow G61 1BD, UK; 6 Institute of Cancer Sciences, University of Glasgow, Glasgow G61 1QH, UK

**Keywords:** oncogenic KRAS, cell competition, pancreas, EphA2, pancreatic cancer, epithelial tissue, homeostasis, E-cadherin, PanINs, early tumorigenesis

## Abstract

As we age, our tissues are repeatedly challenged by mutational insult, yet cancer occurrence is a relatively rare event. Cells carrying cancer-causing genetic mutations compete with normal neighbors for space and survival in tissues. However, the mechanisms underlying mutant-normal competition in adult tissues and the relevance of this process to cancer remain incompletely understood. Here, we investigate how the adult pancreas maintains tissue health *in vivo* following sporadic expression of oncogenic *Kras* (*KrasG12D*), the key driver mutation in human pancreatic cancer. We find that when present in tissues in low numbers, KrasG12D mutant cells are outcompeted and cleared from exocrine and endocrine compartments *in vivo*. Using quantitative 3D tissue imaging, we show that before being cleared, KrasG12D cells lose cell volume, pack into round clusters, and E-cadherin-based cell-cell adhesions decrease at boundaries with normal neighbors. We identify EphA2 receptor as an essential signal in the clearance of KrasG12D cells from exocrine and endocrine tissues *in vivo*. In the absence of functional EphA2, KrasG12D cells do not alter cell volume or shape, E-cadherin-based cell-cell adhesions increase and KrasG12D cells are retained in tissues. The retention of KRasG12D cells leads to the early appearance of premalignant pancreatic intraepithelial neoplasia (PanINs) in tissues. Our data show that adult pancreas tissues remodel to clear KrasG12D cells and maintain tissue health. This study provides evidence to support a conserved functional role of EphA2 in Ras-driven cell competition in epithelial tissues and suggests that EphA2 is a novel tumor suppressor in pancreatic cancer.

## Highlights

• Competition with normal cells clears KRASG12D cells from adult pancreas
• KRASG12D cells are eliminated from acinar, ductal, and endocrine compartments
• Functional EPHA2 is essential to clear mutant cells and preserve tissue health
• Retention of KRASG12D cells accelerates appearance of premalignant lesions

## Graphical abstract

**Figure:**
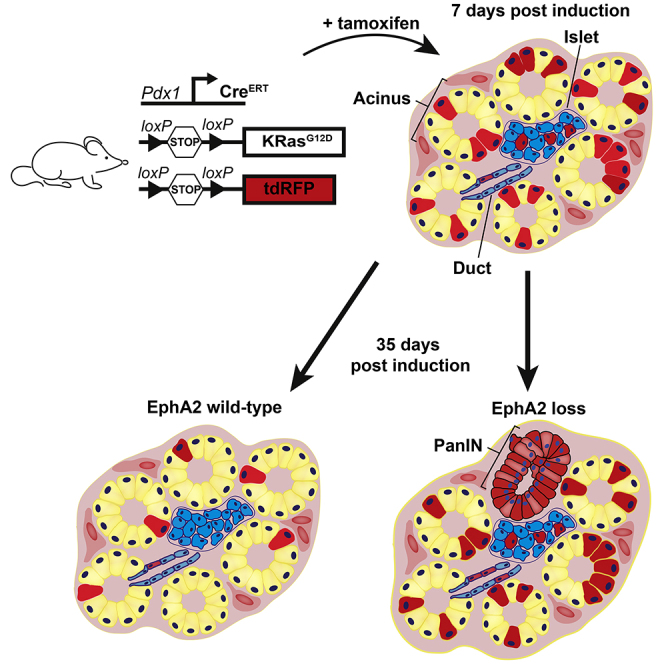

## Teaser

Hill et al. show that KrasG12D-expressing cells are outcompeted from adult pancreas tissues by normal neighbors *in vivo*. Epithelial compartments remodel to eliminate KrasG12D cells in an EphA2-dependent manner. In the absence of EphA2, KrasG12D cells are retained in tissues, increasing the early appearance of premalignant lesions.

## Introduction

Epithelial homeostasis is fundamental to survival and is required to balance the number and fitness of cells that contribute to tissue function. Homeostasis is maintained through distinct processes that dynamically maintain this equilibrium in response to tissue crowding,^1^ damage,^2^ or mutational insult.^3^, ^4^, ^5^, ^6^, ^7^, ^8^, ^9^, ^10^ Retention of excess, mutant, or aberrant cells would impair tissue integrity and promote disease.^11^, ^12^, ^13^ The mechanisms underlying these processes are multifaceted and involve cell competition,^14^ mechanical cues,^15^ and cell plasticity.^16^ Epithelial cells expressing oncogenes compete for space and survival in tissues and are often eliminated via processes that require the presence of normal cells; however, the mechanisms underlying how normal cells sense and eliminate mutant cells remain incompletely understood. We recently identified differential EphA2 signaling as a novel, evolutionary conserved mechanism that drives the segregation and elimination of RasV12 cells in simple epithelia.^10^^,^^17^ EphA2 is a receptor tyrosine kinase of the Eph-ephrin family of cell-cell communication signals that play a general role in regulating cell proliferation and survival and cell-cell adhesion at tissue boundaries, leading to the compartmentalization of cells.^18^ Whether EphA2 is a general regulator of mammalian tissue homeostasis *in vivo* is currently unknown.

The pancreas is composed of functionally distinct compartments of epithelial cells derived from common progenitors.^19^ Exocrine acinar cells produce and secrete digestive enzymes that travel to the gut via ductal networks. Endocrine cells of the islets of Langerhans produce and secrete hormones that regulate blood glucose. Pancreatic cancer arises from activating mutations in oncogenic Kras, and the majority (90%) of human pancreatic tumors develop sporadically from cells carrying *KRAS* mutations;^20^ however, *KRasG12D* mutations alone are insufficient to drive malignancy.^21^^,^^22^ Pancreatic ductal adenocarcinoma (PDAC; the most common form of human pancreatic cancer) develops predominantly from pancreatic intraepithelial neoplasia (PanIN).^23^ The cancer cell of origin in PDAC remains controversial; however, *in vivo* mouse studies indicate that tumorigenesis can develop from cells of exocrine acinar, ducts, and endocrine lineages.^24^^,^^25^ Unlike rapidly proliferating epithelia such as intestine or skin, the adult pancreas is not actively renewing, has limited proliferative capacity during homeostasis, and relies on cell plasticity to regenerate in response to injury.^26^ What is less understood is how the adult pancreas maintains tissue health following mutational insult. Here, we set out to address this question and investigate the requirement of EphA2 in adult pancreas following sporadic, sparse induction of KrasG12D-expressing cells *in vivo*, recapitulating a scenario of sporadic tumorigenesis. Using fluorescence imaging of murine pancreas tissues and quantitative image analysis platforms, we demonstrate that KrasG12D cells are actively cleared from the adult pancreas over time and in an EphA2-dependent manner. In the absence of functional EphA2, mutant cells are retained, accelerating premalignant lesion formation and suggesting that EphA2-driven competition is tumor suppressive.

## Results

### Sparse KrasG12D mutant cells are lost from adult pancreas tissues over time

**Figure 1:**
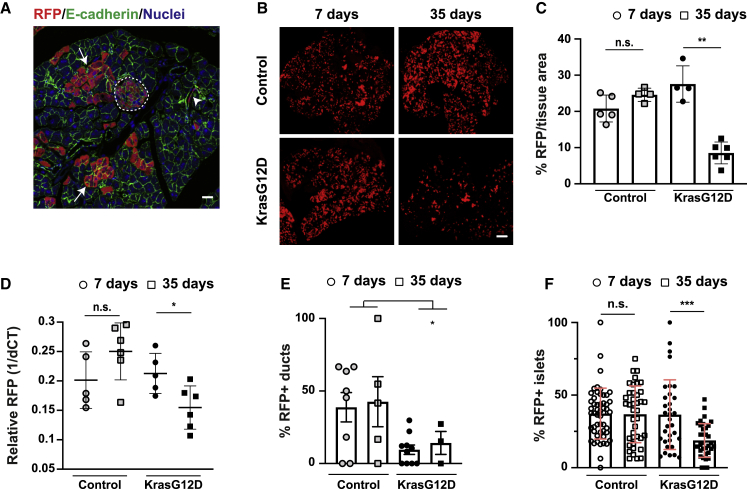
RFP-labeled KrasG12D cells are cleared from pancreas tissue compartments *in vivo* (A) Pancreas tissue (Kras WT) fixed at 7 days p.i. and stained with anti-RFP (red), anti-E-cadherin (green) antibodies, and Hoescht (blue). White arrows, RFP-labeled acinar cells; white arrowhead, RFP-labeled ductal epithelial cells; dashed white line, RFP-labeled islet. (B) RFP fluorescence in tissues harvested from Kras WT (control) or KrasG12D mice. Scale bars, 500 μm. (C) Percentage RFP fluorescence/tissue area over time. Each data point represents average RFP fluorescence per mouse. ^∗∗^p < 0.002, unpaired Student’s t test using Welch correction. Kras WT controls, n = 5 mice (7, 35 days); KrasG12D, n = 4 (7 days), n = 6 mice (35 days). (D) RFP expression in genomic DNA relative to housekeeping gene/tissue/mouse. ^∗^p = 0.024, unpaired Student’s t test using Welch correction; n = 5 (7 days); n = 6 (35 days) mice/genotype. (E) Percentage RFP^+^ ducts in Kras WT control and KrasG12D tissues over time. Data are means ± SEMs. ^∗^p = 0.04, one-way ANOVA. Kras WT controls, n = 8 (7 days), n = 5 mice (35 days); KrasG12D, n = 10 (7 days), n = 3 mice (35 days). (F) Percentage RFP^+^ islet cells/total islet cells in Kras WT control and KrasG12D tissues over time. Data represent mean ± SD islets pooled from n = 4 mice/genotype. ^∗∗∗^p = 0.0008, non-parametric Student’s t test. Kras WT controls, n = 52 islets (7 days), n = 40 islets (35 days); KrasG12D, n = 33 islets (7 days), n = 35 islets (35 days). See also Figures S1 and S2.

We used the pancreas-specific *Pdx1-Cre*^*ERT*^ *LSL-Kras*^*G12D/+*^*; Rosa26*^*LSL-tdRFP*^ (KC; red fluorescent protein [RFP]) mouse and administered a single low dose of tamoxifen to induce *Pdx1*-Cre recombinase in a low number of cells in an otherwise normal epithelium. Experimental controls were *Pdx1-Cre*^*ERT*^*; Rosa26*^*LSL-tdRFP*^ (*Kras* wild type [WT]; control). This approach generated tissues mosaic for *Kras* (and RFP) expression; stochastic RFP labeling was induced in endocrine and exocrine acinar and ductal lineages at low frequency (Figure 1A), ∼20% of tissue (Figure S1A). In contrast, the administration of a high dose of tamoxifen resulted in RFP expression in ∼80% of the tissue (Figure S1A). RFP labeling was comparable in Kras WT and KrasG12D tissues at 7 days post-induction (p.i.) (Figures 1B and 1C). We observed minimal variation in the levels of recombination when sampling from the tail, middle, or head of the pancreas (Figure S1B).

Based on our previous studies,^22^ we reasoned that putative competition between KrasG12D and normal cells in adult tissues would occur over protracted time points. We chose 35 days p.i. as an endpoint and monitored the amount of RFP fluorescence in tissues over time. We found that the proportion of RFP^+^ tissue did not significantly change between 7 and 35 days p.i. in control animals (Figures 1B, upper panels, p = 0.092, and 1C). In contrast, RFP fluorescence significantly decreased over time in KrasG12D tissues (Figures 1B, lower panels, p < 0.002, and 1C), suggesting that KrasG12D cells are cleared from adult tissues over a 4-week period. To validate these findings at the genetic level, we analyzed relative levels of recombined RFP in genomic DNA isolated from pancreas. Consistently, we found that the relative amount of recombined RFP in genomic DNA significantly decreased in KrasG12D tissues (p = 0.024; Figure 1D), whereas levels of recombined RFP remained constant in Kras WT controls (p = 0.13; Figure 1D). Crucially and in contrast to that observed in developing tissues,^22^ the proportion of RFP^+^ tissue remained unchanged over time in adult KrasG12D tissues treated with high-dose tamoxifen (Figures S1C, lower panels, p = 0.5, and S1D), suggesting that the selective loss of RFP^+^ cells from adult KrasG12D tissues is not cell autonomous and requires the presence of normal cells. Consistent with previous reports,^11^ RFP^+^ ducts were significantly less frequent in KrasG12D tissues compared to Kras WT controls (p = 0.04; Figure 1E). The number of RFP^+^ islet cells also significantly decreased over time in KrasG12D tissues (p = 0.0008; Figure 1F). Since exocrine acinar cells are predominately labeled with RFP (Figure 1A), we conclude that KrasG12D cells are outcompeted by normal cells in all epithelial compartments in adult pancreas *in vivo*.

### KrasG12D mutant cells are outcompeted in an EphA2-dependent manner

To determine the mechanisms underlying KrasG12D cell competition, we monitored tissue homeostasis by scoring cell proliferation and cell death events in fixed tissues. Using TUNEL assays (Figure S2A) and immunostaining for cleaved caspase 3 (Figure S2B), we found that apoptosis events were extremely rare in both KrasG12D and Kras WT tissues, and we found no bias in the localization of rare apoptotic cells to RFP^+^ cells (not shown). Apoptotic events remained unchanged over time regardless of genotype (p > 0.1; Figure S2B), suggesting that KrasG12D cells are not triggered to die by apoptosis. We found no evidence of cell senescence in tissues (data not shown). The number of Ki67^+^ cells per tissue area significantly decreased over time in both Kras WT tissues and KrasG12D (p < 0.004; Figure S2C). This is consistent with reports that cell proliferation decreases in aging pancreas tissues over time,^27^^,^^28^ and it implies that changes in cell proliferation rates are unlikely to contribute to the clearance of KrasG12D cells from KC tissues.

**Figure 2:**
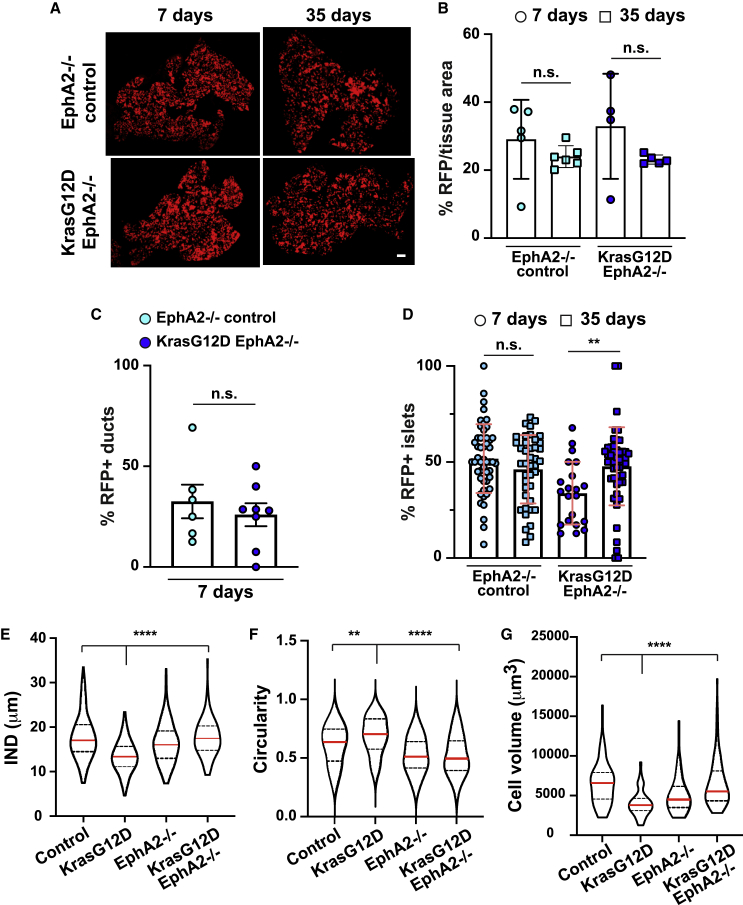
KrasG12D cells are retained in EphA2 knockout tissues (A) RFP fluorescence in EphA2^−/−^ control or KrasG12D/^+^ EphA2^−/−^ tissues. Scale bars, 500 μm. (B) Percentage of RFP fluorescence/tissue area over time. Each data point represents average RFP fluorescence per mouse. EphA2^−/−^ controls, n = 5 (7 days), n = 6 mice (35 days); KrasG12D EphA2^−/−^, n = 4 (7 days), n = 5 mice (35 days). (C) Percentage of RFP^+^ ducts in EphA2^−/−^ control and KrasG12D EphA2^−/−^ tissues at 7 days p.i. EphA2^−/−^ control, n = 6 mice; KrasG12D EphA2^−/−^, n = 8 mice. Data are means ± SEMs. (D) Percentage of RFP^+^ islet cells/total islet cells in EphA2^−/−^ control and KrasG12D EphA2^−/−^ tissues over time. Data represent mean ± SD islets pooled from n = 4 mice/genotype at 7 days, n = 6 mice/genotype at 35 days. n.s., not significant. ^∗∗^p = 0.0035, non-parametric Student’s t test. EphA2^−/−^ controls, n = 49 islets (7 days), n = 41 (35 days) islets; KrasG12D EphA2^−/−^, n = 21 islets (7 days), n = 47 islets (35 days). (E) Violin plots of internuclear distance (IND, μm) between neighboring RFP^+^ cells. (F) Circularity of RFP clusters in tissues. (G) RFP^+^ cell volume (μm^3^). Red line, median. Dashed lines, quartiles. ^∗∗^p < 0.01, ^∗∗∗∗^p < 0.0001, non-parametric Kruskal-Wallis ANOVA with post hoc test comparing KrasG12D to controls and KrasG12D to KrasG12D EphA2^−/−^. Data represent values pooled from 3 mice (*Kras* WT controls, KrasG12D EphA2^−/−^, and EphA2^−/−^ controls) or 4 mice (KrasG12D). See STAR Methods for n numbers. See also Figures S2–S4 and Video S1.

Based on our previous work,^10^^,^^17^ we asked whether EphA2 is required to promote the clearance of KrasG12D cells from adult pancreas tissues *in vivo*. We crossed KC animals onto EphA2 knockout mice, generating *Pdx1-Cre*^*ERT*^; *LSL-Kras*^*G12D/+*^*; Rosa26*^*LSL-tdRFP*^; *Epha2*^*−/−*^ mice (referred to as KCE). *Pdx1-Cre*^*ERT*^; *Rosa26*^*LSL-tdRFP*^; *Epha2*^*−/−*^ (EphA2^−/−^ control) animals were included as controls. Mice homozygous for the targeted *EphA2* mutation are deficient for EphA2 protein^29^ and are therefore knockout for EphA2. By comparing the amount of RFP fluorescence in 7-day tissues, we found no significant difference in the level of recombination between different cohorts (p = 0.15, EphA2^−/−^ versus Kras WT control; p = 0.34, KrasG12D EphA2^−/−^ versus KrasG12D). Similar to Kras WT control tissues, we observed that the proportion of RFP^+^ tissue did not significantly change in EphA2^−/−^ control tissues over time (Figures 2A, top panels, p = 0.57, and 2B). Notably and in contrast to KrasG12D tissues (Figures 1B–1D), RFP fluorescence was not significantly different over time in KrasG12D EphA2^−/−^ tissues (Figures 2A, lower panels, p = 0.29, and 2B; KrasG12D EphA2^−/−^), indicating that RFP^+^ KrasG12D cells are not cleared from tissues depleted of EphA2. Moreover, KrasG12D EphA2^−/−^ and EphA2^−/−^ control 7-day tissues contained similar numbers of RFP^+^ ducts (p = 0.53; Figure 2C), further indicating that KrasG12D cells are cleared from ductal epithelial tissues at very early time points. Notably, the number of RFP^+^ ducts was significantly increased in KrasG12D EphA2^−/−^ tissues compared to KrasG12D tissues (p = 0.028; compare Figure 2C to Figure 1E). RFP^+^ islets also significantly increased in KrasG12D EphA2^−/−^ tissues over time (p = 0.0035; Figure 2D). We observed no significant difference in the number of cleaved caspase-3^+^ cells over time in KrasG12D EphA2^−/−^ tissues (p = 0.18; Figure S2D); however, apoptotic events were significantly higher in younger EphA2^−/−^ tissues (p = 0.021), indicating that EphA2 signaling prevents apoptosis, which is consistent with previous reports.^30^ Cell proliferation events significantly decreased in aging KrasG12D EphA2^−/−^ tissues (p = 0.0035) and in aging EphA2^−/−^ controls (p = 0.045; Figure S2E), similar to that observed in KrasG12D or Kras WT tissues. We conclude that KrasG12D cells are outcompeted from all tissue compartments in an EphA2-dependent manner and independent of changes in global cell proliferation/cell death. Moreover, KrasG12D cells are retained in EphA2-depleted tissues.

### KrasG12D cells shrink and form tightly packed round clusters in an EphA2-dependent manner

Competitive interactions with normal cells induce RasV12 cells to adopt a contractile morphology and segregate from normal neighbors.^4^^,^^10^^,^^17^ To gain insight into KrasG12D cell fate *in vivo*, we examined KrasG12D cell morphology in fixed pancreas tissues using immunofluorescence tomography (IT)^31^^,^^32^ and quantitative image analysis. Fixed serial tissue sections were immunolabeled for E-cadherin and RFP, imaged by confocal microscopy and aligned and stacked to generate 3D reconstructions (Video S1). Individual cells were segmented based on E-cadherin staining. Using internuclear distance measurements between cells in direct contact^10^ and circularity as a readout of cluster roundness, we found that RFP^+^ acinar cells formed significantly more compact (p < 0.0001; Figure 2E) and round (p = 0.0072; Figure 2F) clusters in 7-day KrasG12D tissues compared to Kras WT controls. In addition, RFP^+^ acinar cells significantly decreased in cell volume in KrasG12D tissues compared to Kras WT controls (p < 0.0001; Figure 2G). In contrast, KrasG12D cells no longer formed tightly packed or round clusters (p < 0.0001; Figures 2E and 2F) and did not decrease in cell volume (p < 0.0001; Figure 2G) in KrasG12D EphA2^−/−^ tissues.

Video S1. Visualizing RFP^+^ acinar cells in 3D tissues, related to Figures 2, 4, and 5 Representative movie of stacked confocal images of murine pancreas tissues. Using immunofluorescence tomography (IT) protocols, fixed tissues were cut into serial sections (ribbons), immunostained with anti-E-cadherin (green) and anti-RFP (red) antibodies, and Hoescht (blue) and imaged by confocal microscopy. Images were aligned and stacked to create 3D tissues using Imaris software. Movie depicts KrasG12D (KC) tissue fixed at 7 days post tamoxifen induction.

**Figure 3:**
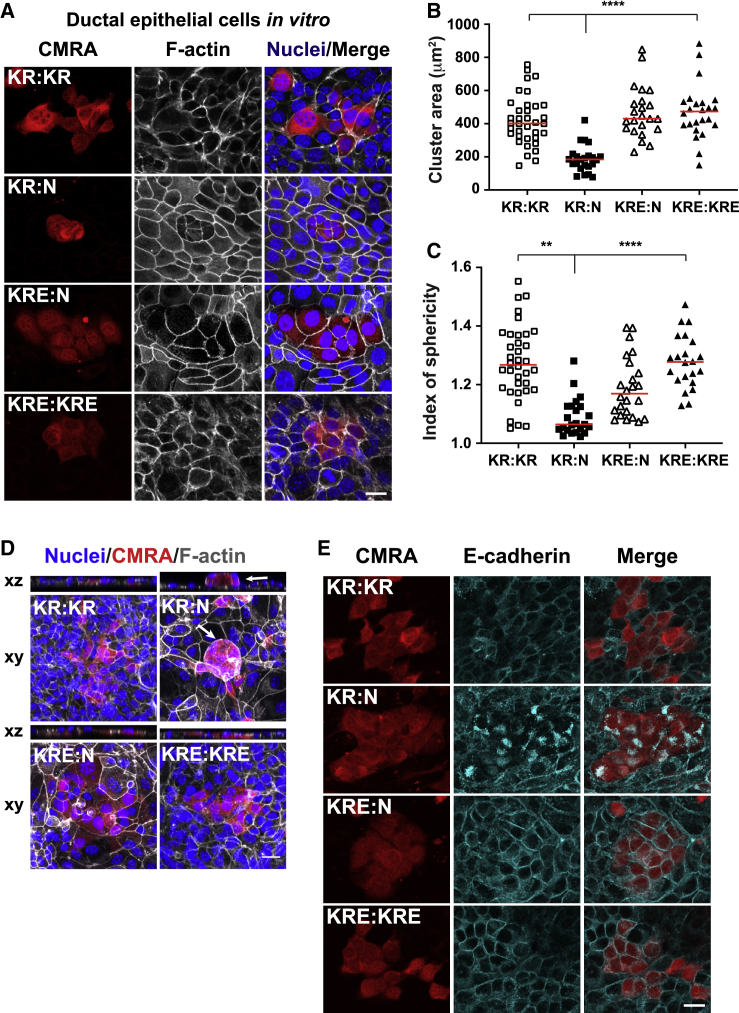
EphA2 expressed on KrasG12D cells drives cell segregation and apical extrusion of mutant cells from normal tissues *in vitro* (A, D, and E) Transformed tumor-derived epithelial cells (KR, KrasG12D; KRE, KrasG12D EphA2^−/−^) prelabeled with cell tracker dye (CMRA, red) and mixed with non-labeled, non-transformed PDECs (N) (KR:N, KRE:N) or non-labeled transformed cells (KR:KR, KRE:KRE) at 1:50 ratios. Cells were fixed at 48 h. (A) Images of pancreatic ductal epithelial cell (PDEC) coculture assays. (D) KrasG12D cells are apically extruded in an EphA2-dependent manner. White arrows label apically extruded cells. (A and D) Gray, F-actin; blue, Hoescht. (E) Cyan, anti-E-cadherin antibodies; blue, Hoescht. Scale bars, 20 μm. (B and C) Scatterplots of (B) transformed cell cluster area (μm^2^) in coculture assays and (C) index of sphericity of normal mutant boundaries. Red lines denote median. Data represent counts from n = 3 repeats. ^∗∗^p < 0.01, ^∗∗∗∗^p < 0.0001, non-parametric Kruskal-Wallis ANOVA with post hoc test comparing KR:N to KR:KR and KR:N to KRE:N. See STAR Methods for n numbers.

To further investigate the requirement of EphA2 in driving these phenotypes, we isolated primary murine pancreatic ductal epithelial cells (PDECs) and applied established coculture assays *in vitro*.^4^^,^^10^ When mixed with non-transformed PDECs at 1:50 ratios, pre-labeled KrasG12D ductal epithelial cells formed tightly packed clusters (Figure 3A; KR:N), that significantly decreased in cell area in a non-autonomous manner (p < 0.0001; Figure 3B; KR:N versus KR:KR). Quantification of the index of sphericity indicated that clusters of KrasG12D cells were significantly more round when surrounded by normal cells (KR:N) compared to KR:KR controls (p < 0.0001; Figure 3C), suggesting that KrasG12D cells separate from normal neighbors via the formation of smooth boundaries. Crucially, KrasG12D cells depleted for EphA2 (KRE cells) no longer adopted a contractile phenotype (Figure 3A; KRE:N), had a significantly higher cluster area when surrounded by normal cells (p < 0.0001; Figure 3B), and segregated less efficiently from normal neighbors compared to KrasG12D cells (p = 0.0052; Figure 3C). We also observed apical extrusion of KrasG12D cells from normal monolayers in an EphA2-dependent manner (Figure 3D, white arrows). Thus, EphA2 expressed on KrasG12D cells is required to promote cell segregation and elimination of KrasG12D pancreatic ductal epithelial cells *in vitro*. Our data show that acinar and ductal epithelial KrasG12D cells shrink and form tightly packed clusters that separate from normal neighbors in an EphA2-dependent manner.

### KrasG12D cells are outcompeted at normal mutant boundaries

To gain further insight into how KrasG12D cells are cleared from tissues, we developed a mathematical model of mutant-normal cell competition. In the model, mutant cells in direct contact with normal cells are always outcompeted (Figure S3A). Simulations of competition between mutant and normal cells revealed that mutant cells are eliminated from the periphery of a mutant cluster, irrespective of cluster size, leading to an overall decrease in the size of the cluster (Figure S3A, bottom panels). In addition, mutant cells are readily eliminated when present in tissues in low numbers (Figure S3B). The model predicts that clusters decrease in size by losing 10% of the mutant-normal cell boundary per day (see STAR methods for details). To examine experimentally mutant-normal competition at the level of cluster size, we segmented global RFP fluorescence data to quantify RFP-labeled clusters in tissues (Figure S3C) and determined the distribution of clusters based on size. Cluster density did not significantly change over time in Kras WT tissues (p = 0.28; Figure S3D). In contrast, cluster density significantly decreased over time in KrasG12D (KC) tissues (p = 0.025; Figure S3D). Consistently, mathematical modeling of mutant cluster distribution based on size predicted a decrease in both cluster density and smaller clusters in 35-day KrasG12D tissues (Figure S3E). Cluster density did not significantly change in EphA2^−/−^ tissues (Figure S3D). Next, we experimentally analyzed the distribution of clusters based on size. In contrast to Kras WT controls (Figure S4A), KrasG12D tissues contained significantly fewer smaller clusters (of <2,000 μm^2^) at 35 days p.i. (Figures S4B and S4E; WT versus KC). In contrast, KrasG12D EphA2^−/−^ (KCE) tissues contained significantly more small clusters at 35 days p.i. compared to KrasG12D tissues at the same time point (Figures S4C and S4E; KC versus KCE), with a size distribution of RFP^+^ clusters in KrasG12D EphA2^−/−^ tissues that is comparable to controls. The distribution of clusters based on size did not significantly change over time in EphA2^−/−^ controls (Figure S4D).

Increased mechanical tension at normal mutant boundaries may promote the efficient elimination of small clusters.^15^^,^^33^ Immunostaining of fixed tissues for F-actin and phosphorylated myosin light chain (Figure S4F) indicate that the actin-myosin cytoskeleton is not enriched at KrasG12D-normal boundaries *in vivo*, suggesting that KrasG12D cells are unlikely to be eliminated via mechanical tension at cluster boundaries. To control for the observed changes in acinar cell morphology *in vivo* (Figures 2E–2G), we quantified the number of RFP^+^ acinar cells per cluster at the single-cell level. This revealed that the number of RFP^+^ cells per cluster significantly decreased in KrasG12D (KC) tissues only, irrespective of cluster size (p = 0.002; Figure S4G). In contrast, RFP^+^ cells per cluster significantly increased in KrasG12D EphA2^−/−^ (KCE) tissues (p = 0.017; Figure S4G). These data support the model and suggest that competition with normal cells leads to a progressive loss of mutant cells from all clusters and an overall decrease in cluster size, in an EphA2-dependent manner.

### EphA2 is required to destabilize E-cadherin-based cell-cell contacts at normal mutant boundaries

**Figure 4:**
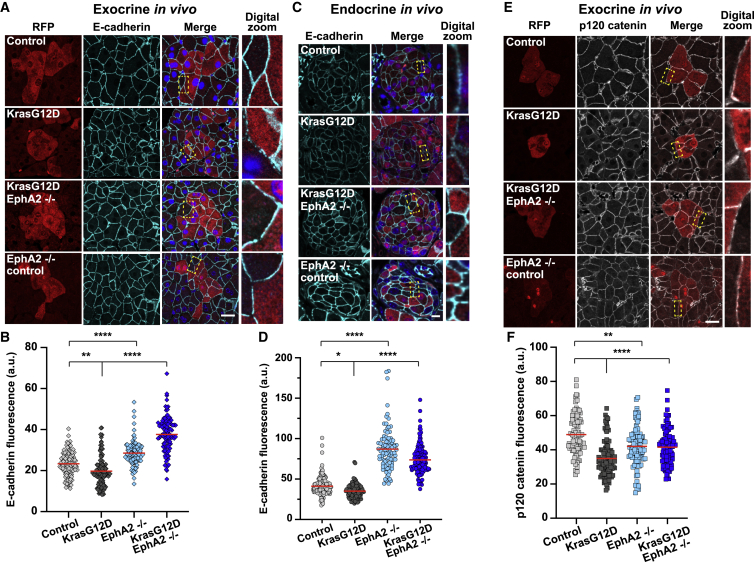
Adherens junctions are remodeled at mutant-normal cell-cell contacts in exocrine and endocrine tissues in an EphA2-dependent manner (A, C, and E) Images of exocrine/endocrine tissues from each genotype fixed at 7 days p.i. and stained with anti-RFP (red), anti-E-cadherin (cyan; A and C), or p120-catenin (gray; D) antibodies and Hoescht (blue). Area in yellow dashed box in merged image: digital enlarged (“digital zoom”) image. Scale bars, 20 μm. See also Video S1. (B, D, and F) Scatterplots of E-cadherin (B and D) or p120-catenin (F) fluorescence at cell-cell interfaces between RFP^+^-RFP^−^ cells in *Kras* WT control, KrasG12D, EphA2^−/−^, or KrasG12D EphA2^−/−^ tissues. Red bar denotes the mean. Data represent cell-cell contacts pooled from n = 3 mice (*Kras* WT controls, KrasG12D EphA2^−/−^, EphA2^−/−^ controls), n = 4 mice (KrasG12D). See STAR Methods for n numbers. ^∗^p < 0.05, ^∗∗^p < 0.005, ^∗∗∗∗^p < 0.0001, non-parametric Kruskal-Wallis ANOVA with post hoc test comparing KrasG12D to control and KrasG12D to KrasG12D EphA2^−/−^, or control to EphA2^−/−^.

We hypothesized that E-cadherin-based cell-cell contacts are remodeled *in vivo* in response to a loss of mutant cell volume and before mutant cell elimination. We immunostained pancreas tissues fixed at 7 days p.i. for endogenous E-cadherin. Focusing on acinar and islet compartments, we found that E-cadherin localizes uniformly at cell-cell contacts in Kras WT tissues (Figures 4A and 4C; control) but appeared weaker and punctate at cell-cell contacts in KrasG12D tissues (Figures 4A and 4C). Quantification of E-cadherin fluorescence at cell-cell contacts between RFP^+^ and RFP^−^ cells revealed that E-cadherin was significantly decreased at RFP^+^/RFP^−^ interfaces in acinar (p = 0.0048; Figure 4B) and islet (p = 0.013; Figure 4D) compartments in KrasG12D tissues compared to Kras WT controls. Similarly, E-cadherin was predominantly intracellular in KrasG12D ductal epithelial cells in a non-cell autonomous manner *in vitro* (Figure 3E; KR:N). In contrast, E-cadherin was significantly enriched at cell-cell contacts between RFP^+^ cells and RFP^−^ neighbors in acinar and islet compartments in KrasG12D EphA2^−/−^ tissues compared to KrasG12D (Figures 4A and 4C, p < 0.0001, Figures 4B and 4D), and was uniformly localized at cell-cell contacts when KRE cells were surrounded by non-transformed cells *in vitro* (Figure 3E). Interestingly, E-cadherin fluorescence was significantly higher at RFP^+^/RFP^−^ boundaries in acinar and islet compartments in EphA2^−/−^ control tissues compared to Kras WT controls (p < 0.0001; Figures 4B and 4D), suggesting that EphA2 regulates E-cadherin localization at cell-cell contacts in a cell-autonomous manner. We speculated that EphA2 may promote E-cadherin turnover at mutant-normal cell-cell contacts. We stained tissues fixed at 7 days p.i. for endogenous p120 catenin, which directly binds to the intracellular domain of E-cadherin and stabilizes cell-cell adhesion by preventing endocytosis.^34^ Focusing on exocrine acinar cells, we found that p120 catenin poorly localized to cell-cell contacts between RFP^+^ and unlabeled cells in KrasG12D tissues compared to Kras WT controls (Figure 4E). Levels of p120 catenin fluorescence at cell-cell contacts were significantly lower at RFP^+^/RFP^−^ cell-cell contacts in KrasG12D tissues compared to Kras WT controls (p < 0.0001; Figure 4F). In contrast, p120 catenin fluorescence was significantly increased at RFP^+^/RFP^−^ cell-cell contacts in KrasG12D EphA2^−/−^ tissues compared to KrasG12D tissues (Figure 4E, p < 0.0001, Figure 4F), suggesting that EphA2 is required to regulate E-cadherin turnover at mutant-normal cell boundaries *in vivo*. p120 catenin fluorescence was significantly decreased in EphA2^−/−^ tissues compared to WT controls (Figure 4F; p = 0.0046), suggesting that the regulation of E-cadherin downstream of EphA2 requires additional regulators.

### Normal cells expand to compensate for the loss of mutant cells

**Figure 5:**
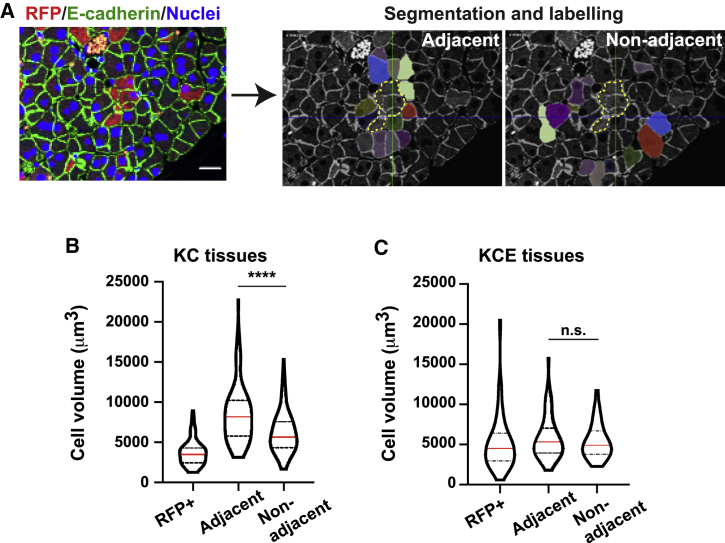
Normal cells directly neighboring KrasG12D cells increase in cell volume *in vivo* in an EphA2-dependent manner (A) Pancreas tissue fixed at 7 days p.i. and stained with anti-RFP (red), anti-E-cadherin (green) antibodies, and Hoescht (blue). Segmentation analysis in 3D labels normal cells (pseudo-colored) neighboring RFP^+^ mutant cells (dashed line). Scale bar, 20 μm. (B and C) Cell volume (μm^3^) of RFP^+^ and unlabeled normal cells, adjacent or non-adjacent to RFP^+^ cells in (B) KrasG12D (KC) and (C) KrasG12D EphA2^−/−^ (KCE) tissues. Red line, median; dashed lines, quartiles. n.s., not significant. ^∗∗∗∗^p < 0.0001, non-parametric Student’s t tests. Data represent volume of individual cells pooled from n = 3 mice KrasG12D EphA2^−/−^ (KCE); n = 4 mice KrasG12D (KC). See STAR Methods for n numbers. See also Video S1.

We postulated that the loss of mutant cells from KrasG12D tissues triggers the compensatory expansion of adjacent WT cells rather than cell proliferation. Using 3D reconstructed tissue datasets, we quantified the cell volume of normal acinar cells either directly adjacent or non-adjacent to RFP^+^ acinar cells (Figure 5A). In KrasG12D tissues (KC), normal cells directly adjacent to RFP^+^ cells had a significantly larger cell volume compared to that of non-adjacent normal cells (p < 0.0001; Figure 5B), suggesting that normal cells in direct contact with KrasG12D cells expand in size. In contrast, the cell volume of normal cells adjacent to RFP^+^ cells was not significantly different from that of non-adjacent cells in KrasG12D EphA2^−/−^ (KCE) tissues (p = 0.46; Figure 5C), suggesting that the expansion of normal cells requires functional EphA2.

### Loss of EphA2 accelerates early development of premalignant lesions

**Figure 6:**
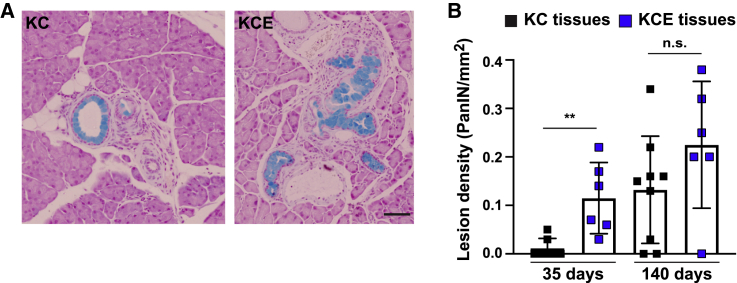
Early appearance of premalignant PanIN lesions in KrasG12D EphA2^−/−^ tissues (A) Pancreas tissues fixed at 140 days p.i. and stained for mucin (blue). Scale bar, 100 μm. (B) Premalignant lesion density (PanIN/mm^2^) in KrasG12D (KC) or KrasG12D EphA2^−/−^ (KCE) tissues over time. Data represent means ± SDs of average number of lesions per tissue area from 4 tissue sections/mouse. n.s., not significant. ^∗∗^p = 0.0029, non-parametric Student’s t test. KrasG12D (KC), n = 7 (35 days), n = 9 mice (140 days); KrasG12D EphA2^−/−^ (KCE), n = 6 (35, 140 days).

Finally, we asked whether the retention of KrasG12D cells in EphA2 knockout tissues correlates with the development of premalignant lesions. While the majority of KrasG12D cells were eliminated by 35 days p.i., a small population of KrasG12D cells consistently remained in KrasG12D tissues (Figures 1B and 1C). Using Alcian blue staining as a marker of mucin-positive PanIN lesions,^35^ we investigated whether the presence of this population of KrasG12D cells in tissues could induce PanIN lesion development. PanIN lesions were rare in KrasG12D (KC) tissues at 35 days p.i. (Figure 6B; 2/7 mice) but were more frequent at 140 days p.i. (Figures 6A and 6B; 7/9 mice), suggesting that non-eliminated KrasG12D cells expand to form lesions over protracted time points. These data are consistent with our previously reported data^22^ and suggest that a subpopulation of KrasG12D cells evades elimination signals to initiate disease. Strikingly, we observed significantly more mucin-positive PanIN lesions in KrasG12D EphA2^−/−^ (KCE) tissues at 35 days p.i. compared to KrasG12D tissues at the same time point (p = 0.0029; Figures 6A and 6B; 6/6 mice). At 140 days, PanIN density was comparable in both KrasG12D (KC) and KrasG12D EphA2^−/−^ (KCE) tissues (Figure 6B). We conclude that the loss of functional EphA2 leads to an increase in the number of KrasG12D cells retained in tissues, which in turn accelerates oncogenic niches and premalignant lesion development at early time points.

## Discussion

Here, we show that cells carrying oncogenic KrasG12D mutations are outcompeted by normal cells in adult pancreas epithelia *in vivo*. We reveal a novel essential role of EphA2 receptor in this process and show that EphA2 is required to remove KrasG12D cells from exocrine acinar, duct, and endocrine compartments *in vivo*. The elimination of KrasG12D cells from each compartment occurs over different timescales, suggesting that different processes may be required to remove KrasG12D cells, depending on tissue location (e.g., local tissue architecture [i.e., a single layer of ductal epithelial cells versus a 3D acinus or islet] and/or EphA2/ephrin A expression). In the absence of functional EphA2, KrasG12D cells are retained and the density of PanIN lesions in tissues increases. Thus, EphA2 is required to maintain pancreas tissue health by promoting the elimination of KrasG12D cells, with the retention of mutant cells accelerating tumor initiation. Our data add to the growing body of evidence^7^^,^^36^, ^37^, ^38^ demonstrating an innate ability of epithelial tissues to prevent tumor initiation following mutational insult.

Eph-ephrin biology in the pancreas is incompletely understood. Both A and B Ephs and ephrins are expressed in the developing pancreas,^39^ and EphB signals regulate pancreas tissue branching, morphogenesis,^40^ and cell fate decisions.^41^ In adult tissues, EphA-ephrinA signals regulate insulin secretion;^42^ however, the role of Eph-ephrin signaling in exocrine tissues is less clear. Eph-ephrins were originally identified as neural guidance molecules that play a crucial role in neurogenesis. Intriguingly, other guidance molecules have been implicated in cell competition in epithelial tissues. In *Drosophila melanogaster*, Slit-Robo signals drive the extrusion of polarity-deficient cells by disrupting E-cadherin,^43^ while semaphorin-plexin signaling promotes the elimination of damaged cells during epithelial wound repair.^44^ We have previously shown in *Drosophila melanogaster* that DEph is required to eliminate RasV12 cells from developing epithelia.^10^ Taken together with our current evidence, these observations suggest that cell-cell communication signals have important functions in maintaining epithelial tissue health and homeostasis.

In general, cell competition induces apoptosis of unfit cells and cell proliferation in surrounding cells, thus ensuring that overall tissue size is maintained.^45^ In the pancreas, apoptotic events are rare, and we found no evidence of the compensatory proliferation of normal cells, suggesting that KrasG12D cell competition does not require the activation of canonical competition signals. However, the targeted inhibition of caspase activity *in vivo* would definitively determine whether mutant cells are eliminated from adult pancreas tissues via apoptosis-dependent signaling. In *D. melanogaster*, cell competition can induce the rapid expansion of “winner” cells immediately neighboring “loser” cells, independent of cell proliferation.^46^^,^^47^ We observe similar phenomena in pancreas tissues *in vivo*: normal cells neighboring KrasG12D cells expand in cell size in response to mutant cells losing cell volume. Mutant and normal cell volumes are not altered in the absence of functional EphA2. Future studies are required to determine the mechanisms underlying how KrasG12D/normal cells lose/gain volume *in vivo* and how EphA2 is contributing to this process.

Our findings imply that EphA2 is a novel tumor suppressor in pancreatic cancer, with the loss of function promoting rapid development of premalignant lesions. Interestingly, loss of EphA2 cooperates with *RAS* mutations to drive skin^48^ and lung^49^ tumorigenesis. Whether EphA2 is required to clear *RAS* mutant cells and preserve tissue health in other epithelial tissues remains to be investigated. Paradoxically, EphA2 is upregulated at the protein level in KRAS-driven tumors^50^, ^51^, ^52^ and is a transcriptional target of RAS-mitogen-activated protein kinase (MAPK) signaling *in vitro*.^10^^,^^53^ Mouse studies indicate that the loss of EphA2 is not essential for primary PDAC tumor development, but it is required for metastasis.^54^ In human tumors, EphA2 expression negatively correlates with PanIN lesions but increases in metastatic tumors;^55^ however, EphA2 is rarely genetically altered in human tumors,^55^ suggesting that protein expression and/or stability is deregulated during tumorigenesis. We propose that tissue context is pivotal to understanding the different roles of EphA2 in tumor biology. Specifically, activation of canonical EphA2 signaling within the context of ephrin ligand-expressing neighbors would drive compartmentalization and expulsion of mutant cells^10^^,^^56^ (and the concomitant degradation of EphA2 protein^57^). In the absence of ligand-expressing neighbors (e.g., EphA2-expressing KRAS-driven tumors), non-canonical signaling of EphA2 would promote metastasis.^54^^,^^58^^,^^59^

Considering that *KRAS* mutations drive all stages of pancreatic cancer^60^ (with codon 12 being the most prevalent, detected in >80% of human tumors^20^), our data imply that KrasG12D mutant cells must be able to override competitive signals to survive in tissues and initiate tumorigenesis in patients. Our study also reveals that a population of KrasG12D cells are never eliminated and go on to initiate and drive premalignant lesion development. Future work is required to determine the mechanisms underlying how rare KrasG12D cells evade competition with normal cells to survive in tissues. A better understanding of the mechanisms by which normal cells outcompete KrasG12D cells could lead to therapies to alter the fitness landscape,^14^ promoting elimination of aberrant cells and decreasing pancreatic cancer incidence. Understanding the mechanisms underlying how KrasG12D cells increase fitness could provide novel insights into how risk factors drive disease.

## STAR★Methods

### Key resources table

**Table:** 

REAGENT or RESOURCE	SOURCE	IDENTIFIER
**Antibodies**		

Mouse anti-E-cadherin	BD Transduction laboratories	Cat#610182; RRID: AB-397581
Mouse anti-p120-catenin	BD Transduction laboratories	Cat#612537; RRID: AB_399834
Rabbit anti-RFP	Creative Diagnostics, Rockland	Cat#H8319; RRID: AB_2428646; Cat#600401-379; RRID: AB_2209751
Rabbit anti-ki67	Abcam	Cat#ab16667; RRID: AB_302459
Rabbit anti-cleaved caspase 3	Cell Signaling Technology	Cat#9661; RRID: AB_2341188
Rabbit anti- phosphorylated myosin light chain 2 (Thr18/Ser19)	Cell Signaling Technology	Cat#3674; RRID: AB_2147464

**Chemicals, peptides, and recombinant proteins**		

Tamoxifen	Sigma-Aldrich/Merck	T5648
Phalloidin	Sigma-Aldrich/Merck	Atto 647N 65906
Cholera toxin	Sigma-Aldrich/Merck	C8052
Bovine pituitary extract	Corning	354123
3,3,5-tri-iodo-L-thyronine	Sigma-Aldrich/Merck	T0281
Epidermal growth factor (EGF) mouse	Corning	354001
Nicotinamide	Sigma-Aldrich/Merck	N3376
Dexamethasone	Sigma-Aldrich/Merck	D4902
Butyl-methyl methacrylate plastic (BMMA) is a mixture of Methyl methacrylate and Butyl methacrylate	Sigma-Aldrich/Merck Sigma-Aldrich/Merck	M55909
		235865
		https://www.nature.com/articles/s41598-018-38232-9#Sec9

**Critical commercial assays**		

TUNEL assay	Abcam	Ab66110
DNAeasy blood and tissue kit	QIAGEN	56304
SyGreen qPCR kit	PCR Biosystems	PB20.11-05

**Experimental models: cell lines**		

Primary murine pancreatic ductal epithelial cells (PDEC)	This paper	N/A

**Experimental models: organisms/strains**		

Mouse: Tg(Pdx1-cre/Esr1^∗^)#Dam	^61^	The Jackson Laboratory 024968
Mouse: Kras < tm4Tyj	^62^	The Jackson Laboratory 008179
Mouse: Gt(ROSA)26Sor < tm1Hjf	^63^	MGI #3696099
Mouse: Epha2tm1Jrui	^29^	The Jackson Laboratory 006028

**Oligonucleotides**		

Genotyping primer. Kras universal CCT TTA CAA GCG CAC GCA GAC TGT AGA	This paper	N/A
Genotyping primer. Kras Mutant AGC TAG CCA CCA TGG CTT GAG TAA GTC TGC A	This paper	N/A
Genotyping primer. Kras wild type GTC GAC AAG CTC ATG CGG GTG	This paper	N/A
Genotyping primer. ROSA RFP Forward AAG GGA GCT GCA GTG GAG TA	^64^	https://doi.org/10.1523/JNEUROSCI.4303-11.2012
Genotyping primer. ROSA RFP reverse AAG ACC GCG AAG AGT TTG TCC	^64^	https://doi.org/10.1523/JNEUROSCI.4303-11.2012
Genotyping primer. RFP Wild type reverse TAA GCC TGC CCA GAA GAC TCC	^64^	https://doi.org/10.1523/JNEUROSCI.4303-11.2012
Genotyping primer. EphA2 Common TGT CAC TTG CGA ACA GTG CT	The Jackson Laboratory	https://www.jax.org/Protocol?stockNumber=006028&protocolID=27262
Genotyping primer. EphA2 Mutant reverse GTG GAG AGG CTT TTT GCT TC	The Jackson Laboratory	https://www.jax.org/Protocol?stockNumber=006028&protocolID=27262
Genotyping primer. EphA2 Wild type reverse CGC TAT CAC ACT CAG CAG GA	The Jackson Laboratory	https://www.jax.org/Protocol?stockNumber=006028&protocolID=27262
Genotyping primers. Pdx-1 Cre Forward: CTG GAC TAC ATC TTG AGT TGC. Reverse: GGT GTA CGG TCA GTA AAT TTG	^65^	https://doi.org/10.1172/JCI65764
Kras recombined. Forward: GTC TTT CCC CAG CAC AGT GC. Reverse: CTC TTG CCT ACG CCA CCA GCT and AGC TAG CCA CCA TGG CTT GAG TAA GTC TGC	This paper	N/A
RFP recombined. Forward: CAA ACT CTT CGC GGT CTT TC. Reverse: CAC CTT GAA GCG CAT GAA CT	This paper	N/A
ApoB. Forward: CAC GTG GGC TCC AGC ATT. Reverse: TCA CCA GTC ATT TCT GCC TTT G	This paper	N/A

**Software and algorithms**		

ImageJ	^66^	https://fiji.sc/
Imaris for Cell biologists 8.0	Bitplane	https://imaris.oxinst.com/
MATLAB code used in mathematical model	This paper	https://github.com/ThomasEWoolley/KRASG12D
Amira for Life and Biomedical sciences	ThermoFisher Scientific	https://www.thermofisher.com/us/en/home/industrial/electron-microscopy/electron-microscopy-instruments-workflow-solutions/3d-visualization-analysis-software/amira-life-sciences-biomedical.html
Zeiss ZEN blue	Zeiss	https://www.zeiss.com/microscopy/int/products/microscope-software.html
GraphPad Prism 8	GraphPad	https://www.graphpad.com/scientific-software/prism/

### Resource availability

#### Lead contact

Further information and requests for resources and reagents should be directed to and will be fulfilled by the lead contact, Catherine Hogan (HoganC@cardiff.ac.uk).

#### Materials availability

This study did not generate new unique reagents.

#### Data and code availability

The code generated during this study are available on the GitHub platform (https://github.com/ThomasEWoolley/KRASG12D).

### Experimental model and subject details

#### Mouse models and primary cell culture

*Pdx1-Cre*^*ERT[61]*^*, LSL-Kras*^*G12D/+[62]*^*, Rosa26*^*LSL-tdRFP[63]*^ *and Epha2*^*−/−[29]*^ mouse lines have all been previously described. Animals were housed in conventional pathogen-free animal facilities and experiments were conducted in accordance with UK Home Office regulations (ASPA 1986 & EU Directive 2010) under the guidelines of Cardiff University Animal Welfare and Ethics Committee. Mice were genotyped by PCR analysis following standard methods. Primer sequences^64^^,^^65^ are detailed in the Key resources table.

Isolation of transformed mouse pancreatic cells was performed as previously described.^22^ Transformed cells were maintained in Dulbecco’s modified Eagle medium (DMEM) supplemented with 10% FBS and 1% penicillin/streptomycin. Normal primary ductal epithelial cells were isolated from pancreata harvested from adult mice of both sexes, as described previously.^67^ Briefly, whole pancreas was mechanically dissociated before digestion in collagenase at 37°C. Following several washes in HBSS supplemented with 5% FBS, tissue was passed through a 40 μm cell strainer. Cells were then digested with trypsin for 5 min before washing to remove any trace of collagenase. The washed cells were then resuspended in PDEC medium (DMEM/F12 with 5% Nu-Serum, 5 mg/ml glucose, 1.22 mg/ml nicotinamide, 0.1 mg/ml soybean trypsin inhibitor, 25 mg/ml bovine pituitary extract, 20 ng/ml EGF, 100 ng/ml cholera toxin, 5 nM 3,3,5-tri-iodo-L-thyronine, 5 mL/L ITS+ culture supplement, 1 mM dexamethasone). Cells were plated on rat collagen I- (Corning) coated 6-well plates.

### Method details

#### Induction of Cre recombinase *in vivo*

Both male and female experimental and control animals were injected at 6-8 weeks of age by intraperitoneal injection of tamoxifen in corn oil. Low frequency expression of RFP was induced via a single intraperitoneal injection of 1 μg/40 g bodyweight (low dose) with widespread recombination (high dose) induced by three injections of 9 mg/40 g over 5 days.^24^ At specified time points the pancreas was harvested and routinely dissected into three segments. Segments were then either fixed in 10% neutral buffered formalin (Sigma Aldrich) overnight at 4°C for IHC/IF, snap frozen in OCT or fixed in 2% paraformaldehyde for immunofluorescence tomography. No statistical method was used to pre-determine sample size. For animal studies, experiments were not randomized, and investigators were not blinded to allocation during experiments. All experiments were reproduced with at least three independent experiments and at least three animals of each genotype.

#### Scoring RFP in tissues

To measure global levels of endogenous RFP fluorescence, five 10 μm thick cryosections were cut from fresh frozen tissues with each slice at least 50 μm apart and immediately imaged. Whole sections were imaged on a Zeiss confocal LSM 710 using tile scans for brightfield and RFP. RFP fluorescence was averaged from five tissue slices (of 50 μm apart) per mouse. Global endogenous RFP fluorescence was calculated as a proportion of total tissue area. Tissue area was quantified from brightfield images and RFP fluorescence was segmented and measured using ImageJ. For cluster analysis, clusters were discretely segmented and quantified in ImageJ and binned on size before normalizing to total tissue area. This produced a density of clusters of different sizes which was plotted in GraphPad. RFP positive ducts and RFP positive acinar cells were scored in tissues fixed and stained using immunofluorescence tomography protocols (described below). RFP positive islets were scored in formalin-fixed paraffin-embedded (FFPE) tissues and immunohistochemistry protocols (described below). Recombined RFP in genomic DNA (gDNA) was measured by qPCR (SyGreen mix; PCR Biosystems) and primer sets (Key resources table). gDNA extraction was carried out from tissues using the DNeasy genomic DNA kit (QIAGEN) following the manufacturer’s instructions. Levels of recombined RFP allele relative to ApoB were analyzed by the delta Ct method.

#### Tissue staining by immunohistochemistry

Histological staining was performed on FFPE sections. For RFP IHC, tissue was fixed in 10% neutral buffered formalin (Sigma-Aldrich) for 24 h at 4°C. Formalin was replaced by 70% ethanol and tissue was processed into wax blocks by standard methods. Sections were cut at 5 μm thickness, dewaxed and rehydrated. For antigen retrieval, tissue sections were incubated in 20 μg/ml Proteinase K (Roche) diluted in TBS/T for 15 min at 37°C. Sections were blocked in 3% H_2_O_2_ (Sigma Aldrich) for 20 min, followed by 5% Normal Goat Serum (NGS) (S-1000, Vector Labs) for 30 min. Anti-RFP (Rabbit, polyclonal) antibody (Rockland) was used at 1:500. Incubation with the primary antibody was performed overnight at 4°C. Secondary antibody ImmPRESS goat anti-Rabbit (MP-7451, Vector Labs) was added at room temperature for 30 min followed by DAB chromogen (Peroxidase substrate kit: SK-4100, Vector Labs) for 3 min. For cleaved caspase 3 and Ki67 IHC, samples were washed in xylene before rehydration in decreasing concentrations of ethanol. After washing, antigen retrieval was carried out using citrate buffer (10mM, pH 6.0) and boiling in a pressure cooker for 5 min (Ki67) or 8 min (CC3). Endogenous peroxidase was blocked by incubation in 0.5% H_2_O_2_ for 20 min (Ki67) or 3% H_2_O_2_ for 10 min (CC3). Sections were blocked in 20% NGS for 30 min (Ki67) or 5% NGS for 60 min (CC3) before incubation in primary antibody overnight (anti-Ki67, 1:50; anti-cleaved Caspase 3, 1:200). Following washing, samples were incubated with biotinylated secondaries (E0432, DAKO) before visualization using Vectastain ABC kit (PK-6100, Vector Lab) and counterstained with DAB. IHC stained tissues were scanned using the Axio Scan Z1 slide scanner (Zeiss, Cambridge, UK), using a 20X magnification, and images were analyzed using the Zeiss Axio Scan Zen software. Quantification of IHC (cells positive for antibody staining, or proportion of RFP positive islet cells) was carried out manually using Zeiss Zen software. RFP positive islets were scored from four tissue sections (50 μm apart) per mouse, n = 4-6 animals/genotype.

For Alcian Blue staining, FFPE tissue sections were dewaxed in xylene and rehydrated in decreasing concentrations of ethanol, before washing in 3% acetic acid for 5 min. Tissue sections were incubated in staining solution (1% Alcian Blue in 3% acetic acid pH 2.5) for 10 min, before extensively washed in 3% acetic acid. Following water rinsing, tissue sections were counterstained using Nuclear Fast Red solution for 10 min (N3020, Sigma, Nuclear Fast Red: 0.1% in 5% aluminum sulfate). Following staining, specimens were rinsed in water, dehydrated and equilibrated into xylene, and mounted with DPX mountant. The entire tissue area was imaged on a Zeiss Axio Scan Z1 slide scanner followed by surface area measurement using Zeiss ZEN software. Alcian Blue positive PanIN lesions per mm^2^ surface area were counted manually.

#### Tissue staining by immunofluorescence

TUNEL staining was carried out on FFPE tissues using a commercially available kit (Abcam) according to the manufacturer’s protocol and Hoescht 33342. Stained tissues were imaged using a Leica DMI600B inverted epifluorescence microscope. For F-actin and phosphorylated myosin light chain (p-MLC) staining, tissue was fixed in 10% neutral buffered formalin overnight at 4°C, washed in PBS and embedded in OCT. Sections of 50 μm were first defrosted at room temperature overnight, washed in PBS and PBS-T (0.03% Triton x100). Sections were blocked with PBS-T 10% FBS over 60 min at room temperature, followed by incubation with primary antibody (rabbit anti-p-MLC; 1:200) or phalloidin (1:1000, Sigma-Aldrich) and Hoescht 33342 solution for 1 h at room temperature. After washing with PBS-T, sections were mounted in Mowiol.

#### Immunofluorescence tomography (IT)

Immunofluorescence tomography (IT) is a high-resolution 3D reconstruction method based on embedding in methacrylate followed by serial sectioning.^32^ Computational alignment of 2D immunostained serial sections produces a 3D volume rendering and was carried out as previously described.^31^^,^^32^ Briefly, fixed tissue was embedded in butyl-methyl methacrylate plastic (BMMA) under UV following dehydration and resin infiltration. Serial 2 μm-thick sections were then rehydrated, and antigens unmasked by boiling in citrate buffer for at least 7 min. Samples were blocked in 5% NGS before incubation in primary antibody overnight at 4°C. Primary antibodies are detailed in Key resources table and were used at 1:500 dilutions. After three PBS washes, samples were incubated for two hours at room temperature in appropriate secondary antibodies (1:200; Life Technologies) before washing, staining nuclei with Hoescht 33342 and mounting in Mowiol. Individual sections were then imaged and aligned semi-automatically using Amira (Version 5.4). See Video S1.

#### Pancreatic ductal epithelial cell (PDEC) co-culture assays

Cell-cell mixing experiments and staining of cells was carried out as described previously.^4^^,^^10^ In brief, one cell population was labeled with CMRA cell tracker dye (ThermoFisher Scientific) as previously described,^4^ before mixing with unlabelled cells at 1:50 ratios. After 48 hours, cells were fixed in 4% PFA for 15 min, before 15 min permeabilization in 0.25% Triton X-100/PBS and blocking in 3% BSA/PBS for 1 hour. Cells were then incubated in anti-E-cadherin antibody (Key resources table), diluted in blocking buffer, overnight at 4°C. The following day, cells were washed three times with PBS and incubated in secondary antibodies (1:200, Life Technologies) and phalloidin (1:200) for 1 h at room temperature. Cells were then mounted in Mowiol and imaged on a Zeiss confocal LSM 710.

#### Mathematical model

We consider two spatial populations denoted by m for mutant and w for wild-type. These two populations interact on a square based grid, so each point is inhabited by at most one tissue type and the position can be specified by a discrete lattice coordinate system.^68^, ^69^, ^70^ Namely, each lattice site is a square of length δ and the ${\left(i,j\right)}^{th}$ lattice site is either occupied by a wild-type tissue (i.e., m(i,j)=0,w(i,j)=1) or a mutant tissue type (i.e., m(i,j)=1,w(i,j)=0). Whenever a mutant cell is next to a wild-type cell the two cell types compete, with the wild-type cell winning at a rate d. Critically, we only consider two cells as neighbors if they share a boundary. Thus, the cells only interact with their north, east, south and west neighbors (Figure S3A).

By considering a general spatial site (i,j) we write down all possible combinations of actions that can occur at that site. Specifically,

$$\begin{array}{cc} & m\left(i\delta ,j\delta \right)+w\left(\left(i+1\right)\delta ,j\delta \right)\overset{d}{\to }w\left(i\delta ,j\delta \right)+w\left(\left(i+1\right)\delta ,j\delta \right),\\ & m\left(i\delta ,j\delta \right)+w\left(\left(i-1\right)\delta ,j\right)\overset{d}{\to }w\left(i\delta ,j\delta \right)+w\left(\left(i-1\right)\delta ,j\delta \right),\\ & m\left(i\delta ,j\delta \right)+w\left(i\delta ,\left(j+1\right)\delta \right)\overset{d}{\to }w\left(i\delta ,j\delta \right)+w\left(i\delta ,\left(j+1\right)\delta \right),\\ & m\left(i\delta ,j\delta \right)+w\left(i\delta ,\left(j-1\right)\delta \right)\overset{d}{\to }w\left(i\delta ,j\delta \right)+w\left(i\delta ,\left(j-1\right)\delta \right). \end{array}$$

The original interaction equations are simulated using a standard “Gillespie” Stochastic Simulation Algorithm (SSA).^71^, ^72^, ^73^ To use a SSA we first need to calculate the propensity, $a_{r}$, of each reaction, r, where the propensity is the probability per unit time that a specific reaction occurs. Further, we calculate the probability per unit time that any reaction occurs, which is the sum of all propensities,

$$a_{0}=\underset{r}{\sum }a_{r}.$$

The SSA proceeds as follows:

1. Define the initial time, t=0, and the final time, $t_{f},$ over which we wish to run the algorithm.
2. Generate two uniformly randomly distributed numbers, $r_{1}$and $r_{2}$, from the interval [0, 1].
3. Calculate all propensity functions, $a_{r}$, and their sum $a_{0}$.
4. Compute the time, τ, when the next reaction takes place, where

$$\tau =\frac{1}{a_{0}}\ln\left(\frac{1}{r_{1}}\right),$$

and update the current time, t:=t+τ.

5. Find the reaction that fires by searching for the integer, j, such that

$$\overset{j}{\underset{i=1}{\sum }}\frac{a_{i}}{a_{0}}\leq j<\overset{j+1}{\underset{i=1}{\sum }}\frac{a_{i}}{a_{0}},$$

and update the populations based on the reaction that fires.

6. If $t\geq t_{f}$then end the simulation, otherwise go to step 2.

This algorithm allows us to accurately simulate a single stochastic trajectory with the correct probabilistic properties. Simulating multiple trajectories allows us to produce probability distributions that provide us with the probability of the system inhabiting a given state at a given time.

To compare the simulations with the data an initial distribution of mutant patches is needed. Once this has been set the patches can be numerically simulated, so that future patch sizes can be predicted. The power law behind the seven-day density-area curve is extracted using a nonlinear least-squares fit algorithm from MATLAB R2019a curve fitting toolbox. Specifically, a curve of the form $y=ax^{b}$ is fitted to the seven-day data from which we obtain the parameter estimates of a=2436(95% CI 2382−2491) andb=−1.067(95% CI −1.071–−1.063). Upon fixing δ=1μm several different mutant patch sizes are simulated with densities provided by the power law. Namely, if the area of a patch is x then its density was y. During a simulation, the mutant patch sizes shrink as the mutant cells are out competed by the wild-type cells. The simulations are all run for a time, t=35 days, and the competition rate d was chosen such that the final simulated curve provided a nonlinear least-squares best fit to the 35-day data.

### Quantification and statistical analysis

#### Image analysis

Inter-nuclear distance (IND) was calculated by measuring the distance between the center of nuclei of neighboring cells in 3D projections using Imaris (Bitplane, Version 8.0). Nuclei were initially segmented using ‘spots’ before measuring from the center of each spot. To measure cluster area and cell volume, cells were first segmented using Amira. To measure cluster area, index of sphericity and circularity score, clusters were manually segmented using Fiji^66^ (ImageJ, Version 2.0) and calculated as previously described.^10^ Quantification of fluorescent signal for E-cadherin and p120 catenin regions of interest was carried out in ImageJ. Images were taken under the same exposure time and settings and the mean signal intensity was measured

#### Statistical tests

Statistical analyses were performed using GraphPad Prism (v8.0). Normally distributed data, as determined by the Shapiro-Wilke test were analyzed using unpaired Student’s t tests with Welch correction. Data that were not normally distributed were compared using non-parametric Mann Whitney U two-tailed t tests. To compare more than two groups, we used ANOVA (Kruskal-Wallis) tests. Post hoc (Dunn’s multiple comparisons) tests were performed to determine statistical significance between groups. Specifically, we compared data from KrasG12D tissues to Control, and to KrasG12D EphA2−/− tissues. Where relevant, comparisons were also made between Controls and EphA2−/− data. A p value of < 0.05 was taken as significant. No statistical method was used to pre-determine sample size. Definition of *n* defined in the figure legends. Also, Figure 2: (E) *Kras* wild-type control: n = 184 cells; KrasG12D: n = 218 cells; EphA2−/− controls: n = 78 cells; KrasG12D EphA2−/−: n = 162 cells. (F) *Kras* wild-type control: n = 102 clusters; KrasG12D: n = 104 clusters; EphA2−/− controls: n = 78 clusters; KrasG12D EphA2−/−: n = 90 clusters. (G) *Kras* wild-type control: n = 87 cells; KrasG12D: n = 89 cells; EphA2−/− controls: n = 60 cells; KrasG12D EphA2−/−: n = 113 cells. Figure 3: (B) KR:KR: n = 23; KR:N: n = 36; KRE:N: n = 23; KRE:KRE: n = 24 clusters. (C) KR:KR: n = 23; KR:N: n = 36; KRE:N: n = 24; KRE:KRE: n = 22 clusters. Figure 4: Data represent cell-cell contacts pooled from n = 3 mice (*Kras* wild-type controls, KrasG12D EphA2−/−, EphA2−/− controls), n = 4 mice (KrasG12D). (B) Control: n = 111; KrasG12D: n = 106; EphA2−/− control: n = 98; KrasG12D EphA2−/−: n = 96. (D) Control: n = 152; KrasG12D: n = 100; EphA2−/− control: n = 90; KrasG12D EphA2-/: n = 138. (F) Data represent cell-cell contacts pooled from 3 mice/genotype. Control: n = 92; KrasG12D: n = 111; EphA2−/− control: n = 96; KrasG12D EphA2−/−: n = 93. Figure 5: Data represent volume of individual cells pooled from n = 3 mice KrasG12D EphA2−/− (KCE); n = 4 mice KrasG12D (KC). KrasG12D (KC) tissues: n = 101 RFP positive, n = 91 adjacent normal, n = 84 non-adjacent normal. KrasG12D EphA2−/− (KCE): n = 113 RFP positive, n = 61 adjacent normal, n = 61 non-adjacent normal.

## Supplemental information

Document S1. Figures S1–S4

Document S2. Article plus supplemental information
