# Supplementary material for: EPHA2-dependent outcompetition of KRASG12D mutant cells by wild-type neighbors in the adult pancreas
Source: Curr Biol. 2021 Jun 21;31(12):2550–2560.e5. doi: 10.1016/j.cub.2021.03.094 (PMC8231095; doi:10.1016/j.cub.2021.03.094)
Supplement: Document S1. Figures S1–S4 [file mmc1.pdf]

Current Biology, Volume *31*

Supplemental Information

EPHA2-dependent outcompetition

of KRASG12D mutant cells by wild-type

neighbors in the adult pancreas

William Hill, Andreas Zaragkoulias, Beatriz Salvador-Barbero, Geraint J. Parfitt, Markella Alatsatianos, Ana Padilha, Sean Porazinski, Thomas E. Woolley, Jennifer P. Morton, Owen J. Sansom, and Catherine Hogan

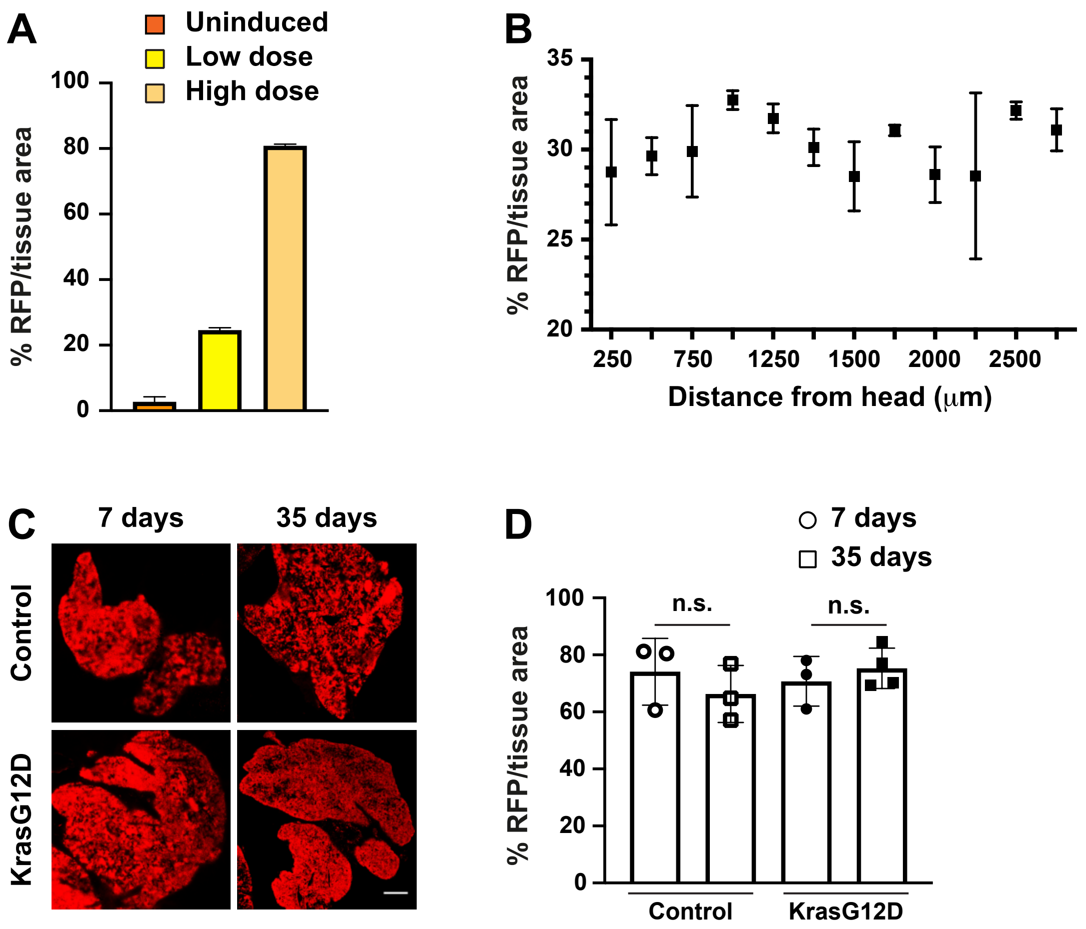

Figure S1: Clearance of KrasG12D cells occurs throughout the pancreas and requires the presence of normal cells. Related to Figure 1. (**A**) Bar graph depicting proportion of endogenous RFP fluorescence per tissue area in Kras wild type control tissues harvested at 7 days p.i following no tamoxifen (uninduced; orange bar), low dose (yellow bar) or high dose (beige bar) tamoxifen. Data represent mean +/- s.d. from 2 mice/treatment. (**B**) Scatter plot showing percentage endogenous RFP fluorescence per tissue area in Kras wild type control tissues harvested at 7 days p.i. Data represent mean +/- s.d. of fluorescence averaged from two mice. Tissue sections were sampled every 250 μm from the head to the tail of the pancreas. (**C**) Representative stitched confocal tile scan images of fresh frozen murine pancreas tissues showing endogenous RFP fluorescence. Tissues were harvested from control (Kras wild type) or KrasG12D at 7 days or 35 days p.i. following high dose of tamoxifen. Scale bar, 500 μm. (**D**) Bar graph showing percentage endogenous RFP fluorescence per tissue area in control (Kras wild type) or KrasG12D tissues, harvested at 7 days or 35 days p.i. following high dose of tamoxifen. Data are mean +/- s.d. of RFP fluorescence per tissue area. Each data point represents average RFP fluorescence per mouse. n.s.=not significant. n=3 mice for control (7 days, 35 days); n=3 mice KrasG12D (7 days) and n=4 mice KrasG12D (35 days).

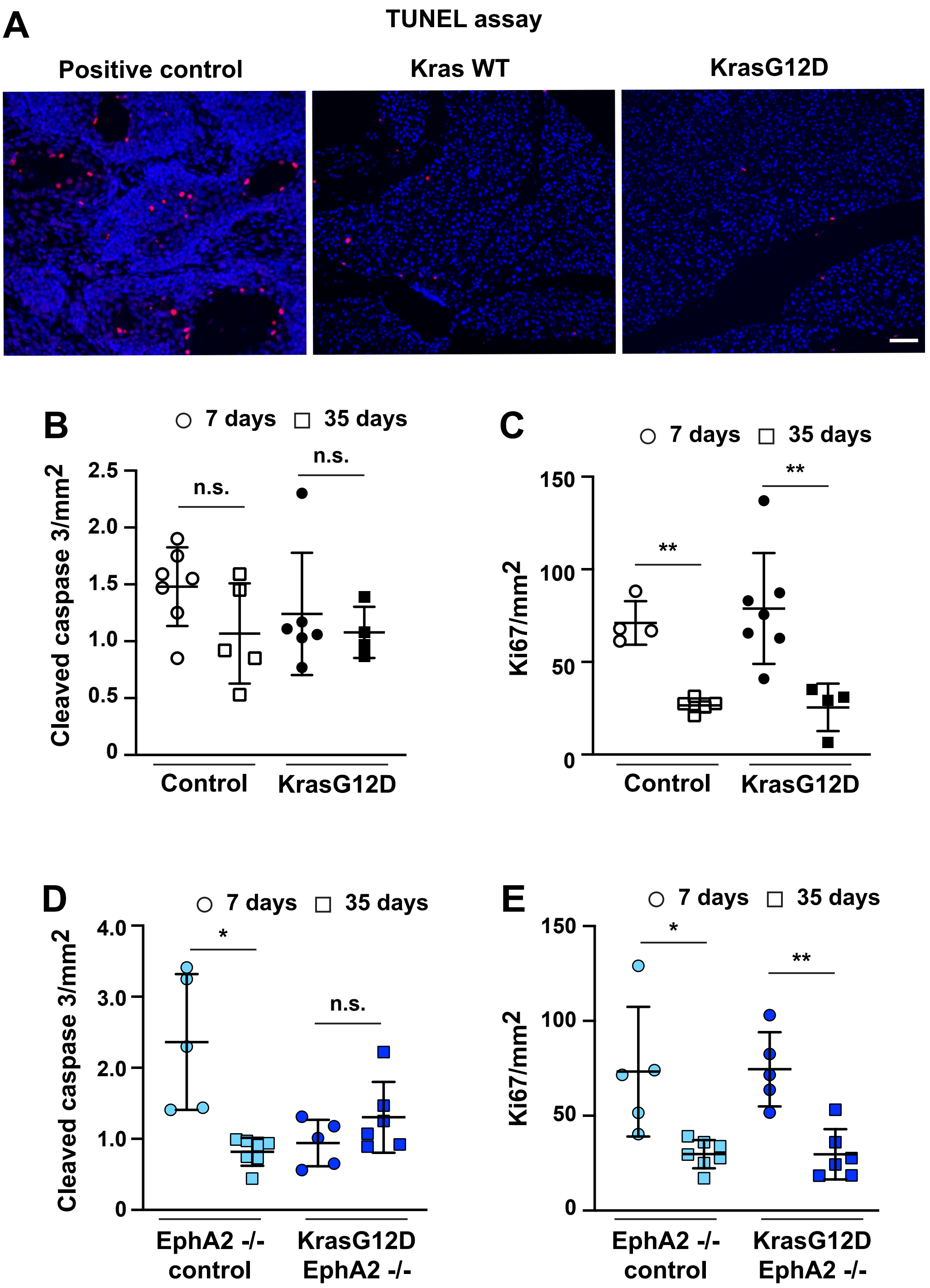

Figure S2: Cell death and cell proliferation events in adult pancreas tissues following low dose tamoxifen induction. Related to Figures 1, 2. (**A**) Representative epifluorescence images of TUNEL assays. Positive control: mouse mammary tumour tissue; Kras WT: Kras wild type control. Kras WT and KrasG12D tissues were fixed at 7 days p.i. following low dose tamoxifen. Scale bar, 100 μm. (**B**-**E**) Scatter plots showing number of cells positive for (**B**) cleaved caspase 3 or (**C**) Ki67 positive cells per tissue area in control (Kras wild type) and KrasG12D harvested at 7 days and 35 days p.i. Data

represent mean +/- s.d. n.s.=not significant. **p<0.004, unpaired Student t tests with Welch correction. For (B), Control: n=7 (7 days) and n=5 (35 days) mice; KrasG12D: n=6 (7 days) and n=4 (35 days) mice. For (C), Control: n=4 (7 days) and n=5 (35 days) mice; KrasG12D: n=7 (7 days) and n=4 (35 days) mice. Scatter plots showing number of cells positive for (**D**) cleaved caspase 3 or (**E**) Ki67 positive cells per tissue area in EphA2$^{-/-}$ control (*Kras* WT) and KrasG12D EphA2-/- tissues harvested at 7 days and 35 days p.i. Data represent mean +/- s.d. n.s.=not significant. (D) *p=0.021, non-parametric Student t Test. EphA2-/- control: n=5 (7 days) and n=7 (35 days) mice; KrasG12D EphA2-/-: n=5 (7 days) and n=6 (35 days) mice. (E) *p=0.045, **p=0.0035, unpaired Student t tests with Welch correction. EphA2-/- control: n=5 (7 days) and n=7 (35 days) mice; KrasG12D EphA2-/-: n=5 (7 days) and n=6 (35 days) mice.

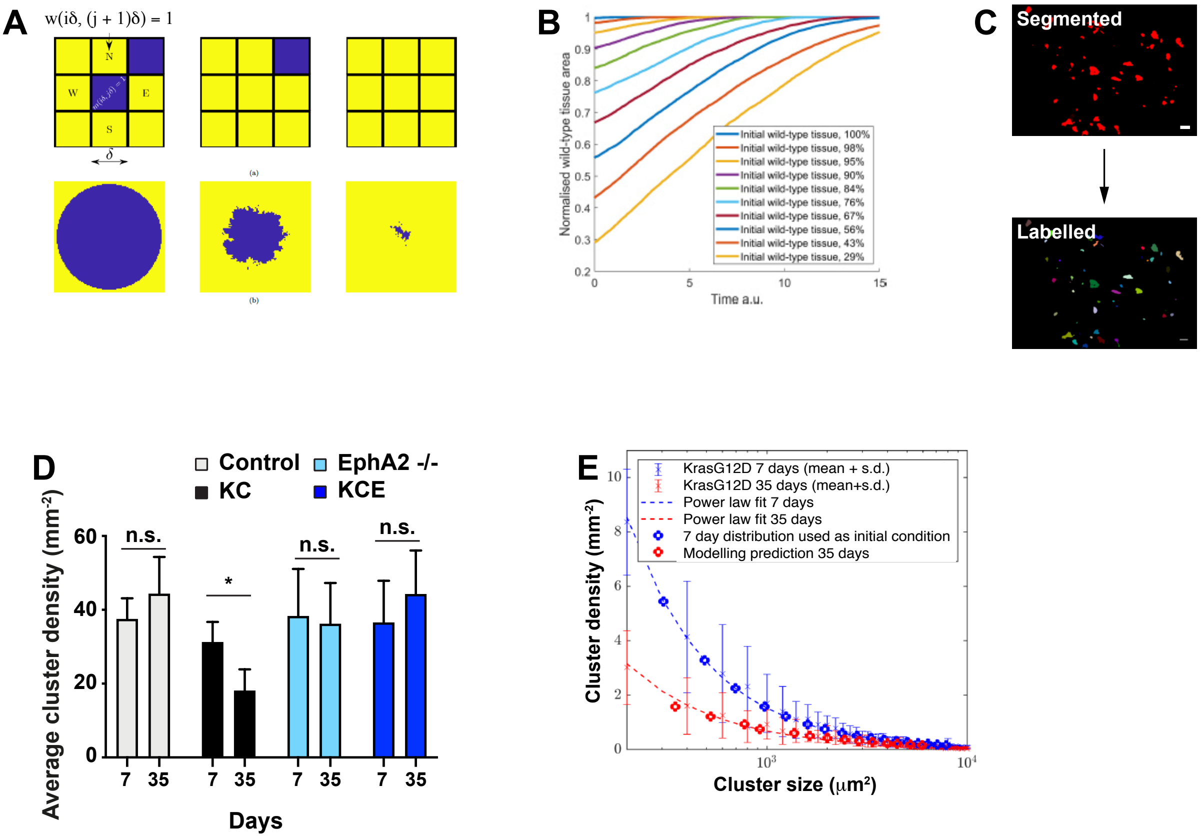

Figure S3: Normal-mutant competition leads to a reduction in mutant cluster size. Related to Figures 1, 2. (**A**) Schematic of mathematical model illustrating dynamics of normal-mutant cell interactions with normal cells in yellow and mutant cells in blue. In the model, mutant cells are always outcompeted by normal cells. Top panels: mutant cell with four neighbours (middle) are eliminated more readily than mutant cells with fewer normal neighbours (blue square, top right). Lower panels: simulation of the model (100x100 grid; mutant cells in blue occupy circle with radius of 50) over three time points. (**B**) Simulations of 100x100 grid as in (A, lower panels) with the radius of mutant cells altered. The initial proportion of wild-type tissue increases as the proportion of mutant tissue decreases. (**C**) Segmented RFP fluorescence labels individual clusters (pseudo-colour). Scale bar, 500 μm. (**D**) Average cluster density/tissue area (mm^{-2}) over time. n.s. = not significant. *p=0.025, unpaired Student t test using Welch correction. (**E**) Frequency distribution curves of KrasG12D clusters of varying size (μm^2) over time. Blue lines/points: KrasG12D cluster density at 7 days; Red lines/points: KrasG12D cluster density at 35 days. Dashed lines: statistical fit using power law equation. Cluster distribution data for 7-day tissues (Figure S3D) was used as an initial condition to predict 35-day curve.

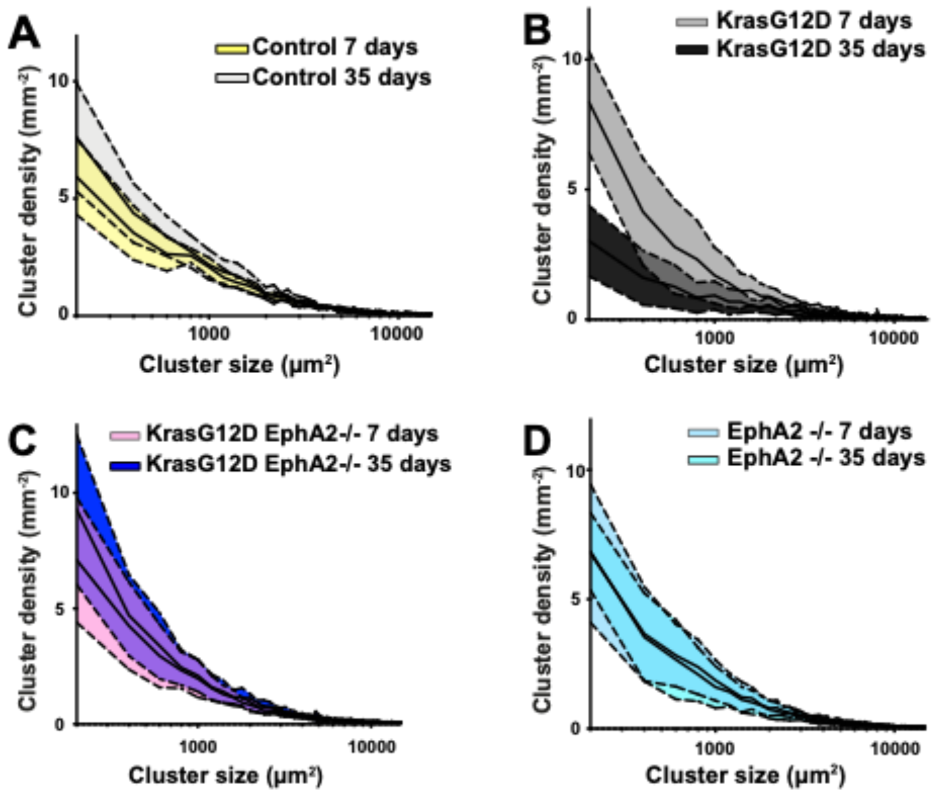

E

Cluster size (μm^2)	*p-value* WT v KC	KC v KCE
200	*0.0095*	*0.0094*
400	*0.0121*	*0.0125*
600	*0.0067*	*0.0254*
800	*0.0044*	*0.0057*
1000	*0.0381*	*0.016*
1200	*0.01*	*0.0211*
1400	*0.0194*	*0.0313*
1600	*0.019*	*0.0047*
1800	*0.0144*	*0.0133*
2000	*0.0357*	0.0823
2200	0.0907	0.0603
2400	*0.0095*	*0.0303*
2600	*0.0217*	*0.0168*
2800	*0.0317*	*0.0318*
3000	0.1251	0.069

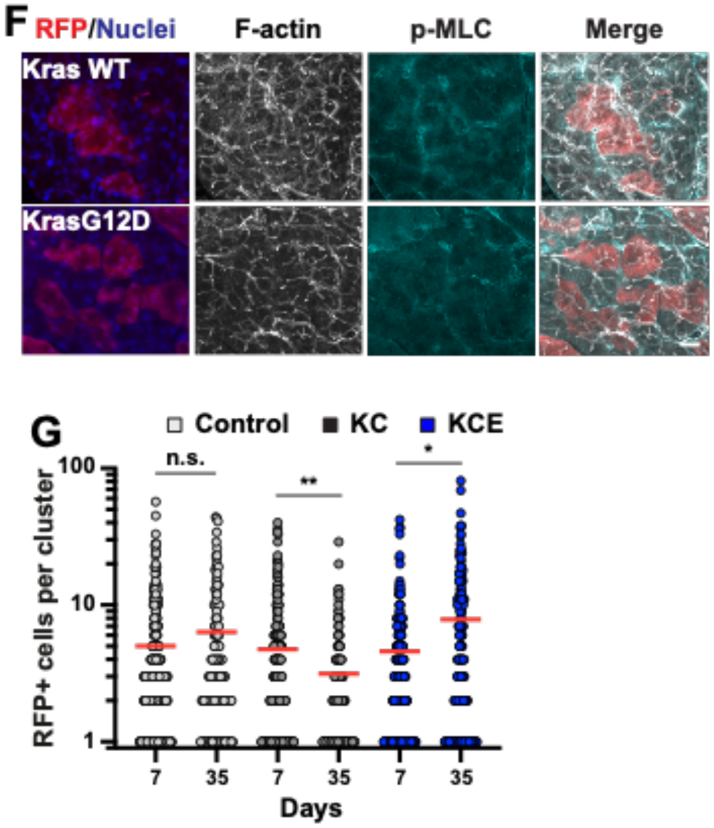

Figure S4: Small clusters of RFP positive cells are less frequent in KC tissues over time in an EphA2-dependent manner. Related to Figures 1, 2. (**A**)-(**D**) Frequency distribution graphs of RFP positive clusters of varying size (mm^{-2}). (**A**) Cluster

density in Kras wild-type control tissues does not vary between 7 days (yellow curve) and 35 days (light grey curve). (**B**) Density of small clusters (<2000μm^2) decreases in KrasG12D tissues at 35 days (black curve) compared to 7 days (grey curve). Cluster density in (**C**) KRasG12D EphA2-/- tissues does not vary between 7 days and 35 days, or in (**D**) EphA2-/-control tissues. Data are mean +/- s.d. of minimum of 12000 clusters per genotype pooled from n=5 mice (7 days and 35 days) Kras wild type controls; n=4 mice (7 days) and n=6 mice (35 days) for KrasG12D; n=5 mice (7 days) and n=6 mice (35 days) for EphA2-/- controls; n=4 mice (7 days) and n=5 mice (35 days) for KrasG12D EphA2-/-. (**E**) Table of p values comparing clusters of different sizes between *Kras* wild-type (WT) and KrasG12D (KC), or KC and KrasG21D, EphA2-/- (KCE) tissues. Data were compared using unpaired Student t test with Welch correction or non-parametric Student t test, depending on results of normality tests. p<0.05 was taken as significant. Values labelled in blue indicate significance. (**F**) Confocal images of pancreas tissues harvested from Kras wild type (WT) or KrasG12D tissues at 7 days p.i. Fixed tissues were stained for F-actin (grey) and anti-phosphorylated myosin light chain (p-MLC; cyan). Endogenous RFP labels recombined cells. Hoescht labels nuclei. Images are maximum projections of z stacks. Scale bar, 20 μm. (**G**) Number of RFP positive acinar cells per cluster. Red bar denotes mean. Data represent values pooled from 3 mice (Kras wild type controls, KrasG12D EphA2-/-, EphA2-/- controls) or 4 mice (KrasG12D). n.s. = not significant. *p=0.017, **p=0.002, non-parametric Student t tests. In (E), (F), (G) Control: Kras wild type control; KC: KrasG12D; KCE: KrasG12D, EphA2-/-.
